# Supplementary material for: Household rat infestation in urban slum populations: Development and validation of a predictive score for leptospirosis
Source: PLoS Negl Trop Dis. 2021 Mar 3;15(3):e0009154. doi: 10.1371/journal.pntd.0009154 (PMC7959339; doi:10.1371/journal.pntd.0009154)
Supplement: S1 Table — (DOC) [file pntd.0009154.s001.doc]

**Supplementary Table 1**: Rodent-related and environmental characteristics among 95 and 67 case households of development and validation groups in Salvador, Brazil.

| **Household characteristics** | **Development***  **(*n* = 95)** | **Validation***  **(*n* = 67)** |  |
| --- | --- | --- | --- |
|  | No. (%) or median (IQR)† | | ***P***‡ |
| **Demographics** |  |  |  |
| No. of inhabitants | 4 (3-5) | 3 (2-5) | - |
| Male sex | 2 (1-2) | 1 (1-2) | - |
| Per capita income, US$/d | 2.6 (1.3-4.1) | 3.1 (2.1-5.2) | - |
| **Premise type and details**§ |  |  |  |
| Residential use only§ | 92 (97) | 63 (94) | - |
| Borders on a vacant lot | 18 (19) | 5 (6) | - |
| Open sewer <10m distance | 30 (32) | 24 (36) | - |
| Borders on an abandoned house | 22 (23) | 13 (20) | - |
| **Access to food sources**§ |  |  |  |
| Exposed garbage§ | 78 (82) | 60 (89) | - |
| Animal food§ | 45 (47) | 31 (46) | - |
| Other food & plants§ | 64 (67) | 39 (58) | - |
| Open stores of human food | 52 (55) | 27 (40) | - |
| **Access to water**§ |  |  |  |
| Standing water§ | 24 (25) | 20 (30) | - |
| Leaks§ | 34 (36) | 27 (40) | - |
| **Harborage for rodents**§ |  |  |  |
| Abandoned vehicles§ | 1 (0) | 0 (0) | - |
| Abandoned appliances§ | 94 (99) | 56 (87) | <0.01 |
| Lumber/clutter on ground§ | 67 (70) | 44 (65) | - |
| Other large rubbish§ | 44 (46) | 35 (52) | - |
| Outbuildings/Privies§ | 19 (20) | 20 (30) | - |
| Dilapidated fences & walls§ | 19 (20) | 18 (27) | - |
| Plant-related§ | 75 (79) | 56 (83) | - |
| Bushes or shrubbery | 42 (44) | 28 (42) | - |
| Ornamental plants | 65 (68) | 54 (81) | - |
| Presence of exposed earth | 61 (64) | 45 (67) | - |
| Built on earthen slope¶ | 50 (53) | 34 (51) | - |
| **Entry/Access**§ |  |  |  |
| Structural deficiencies§ | 64 (67) | 44 (65) | - |
| Hole(s) in roof | 50 (52) | 30 (45) | - |
| Hole(s) in wall | 29 (30) | 21 (31) | - |
| Hole(s) in floor | 19 (20) | 26 (39) | <0.05 |
| Un-plastered walls# | 64 (67) | 41 (61) | - |
| **Rodent active signs**§ |  |  |  |
| Active signs§ | 60 (63) | 35 (52) | - |
| Rodent burrows | 53 (56) | 22 (33) | <0.01 |
| Rodent runs | 33 (35) | 20 (30) | - |
| *R. norvegicus* feces | 28 (29) | 18 (27) | - |
| *R. rattus* feces | 0 (0) | 1 (1) | - |
| *M. musculus* feces | 1 (1) | 2 (2) | - |
| **Domestic animals** |  |  |  |
| Dogs | 39 (45) | 27 (40) | - |
| Cats | 13 (13) | 6 (9) | - |
| Chickens | 13 (13) | 9 (13) | - |

* Case households in which laboratory-confirmed cases of leptospirosis resided during the periods January 2007 – December 2008 (development cohort) and January 2009 – December 2009 (validation cohort).

† Median and inter-quartile range (IQR) values are shown for continuous variables.

‡ Values are not shown for non-significant associations in matched analyses.

§ Categories and variable defined in the CDC form (Centers for Disease Control and Prevention, 2006).

¶ Presence of exposed earth slope (>45º) within 10m of the household.

# Walls composed of exposed bricks without external application of stucco or plastering.
