# Supplementary material for: Household rat infestation in urban slum populations: Development and validation of a predictive score for leptospirosis
Source: PLoS Negl Trop Dis. 2021 Mar 3;15(3):e0009154. doi: 10.1371/journal.pntd.0009154 (PMC7959339; doi:10.1371/journal.pntd.0009154)
Supplement: Attachment — Submitted filename: 31.12.20 When you are ready to resubmit.docx [file pntd.0009154.s002.docx]

Dear editors, PLoS Neglected Tropical Diseases

Here, we submit the revised version of the manuscript “Household Rat Infestation in Urban Slum Populations: Development and Validation of a Predictive Score for Leptospirosis”. The revised version has taken into account the comments and observations presented by the reviewers, to whom we would like to say thanks for their time and effort diverted into their contributions. Comments are addressed point-by-point after this letter.

Sincerely,

The authors.

Methods
-Are the objectives of the study clearly articulated with a clear testable hypothesis stated?

-Is the study design appropriate to address the stated objectives?

-Is the population clearly described and appropriate for the hypothesis being tested?

-Is the sample size sufficient to ensure adequate power to address the hypothesis being tested?

-Were correct statistical analysis used to support conclusions?

-Are there concerns about ethical or regulatory requirements being met?

Reviewer #1

Line 114:  You mention that the MAT was used for diagnosis of clinical cases. Can you confirm that only reactors to rat-associated serovars were included?

*The serovar panel used for the MAT test includes serovars besides rat-associated serovars. Our panel has 26 serovars including the most common Leptospira species in Salvador,* *L. interrogans serogroup Icterohaemorrhagiae serovar Copenhageni (strain Fiocruz L130) (Ko et al). All cases in this study presented higher titers against* *strain Fiocruz L130 for which the major reservoir in the city of Salvador is Rattus norvegicus.*

*We have included sentences in the methodology section and in the result section clarifying this point.*

*Ko AI, Galvão Reis M, Ribeiro Dourado CM, Johnson WD Jr, Riley LW; Salvador Leptospirosis Study Group. Urban epidemic of severe leptospirosis in Brazil. Lancet. 1999;354:820–5. https://doi.org/10.1016/S0140-6736 (99)80012-9*

Line 125: Three or more nights is not intuitive as a definition for permanent household members. Can you justify why that number was chosen? at least 50% occupation

*It responds as residents who spend approximately 50% of the time in the household environment. This definition has been used consistently as a proxy of individual exposures in the household environment in all epidemiological studies of leptospirosis in the city of Salvador and we kept here in order to compare with previous findings. The reason behind this definition is that frequently, residents from slum areas need to sleep outside of their homes for work reasons. By adopting this definition we aimed to include residents who will be exposed to household related risk factors and avoid others spending only weekends at the household.*

Line 152: I see there is a reference supporting this survey tool but can you provide the survey as a supplementary file or link to where a copy is publicly available?

*The survey tool is available as part of the pertinent reference. Is listed as Table S1. English and Portuguese description of variables included in the modified exterior inspection form, adapted from the CDC manual in the following link* [*https://www.ncbi.nlm.nih.gov/pmc/articles/PMC4256176/*](https://www.ncbi.nlm.nih.gov/pmc/articles/PMC4256176/)

*Given that this form has been already published we prefer to avoid to include it in the current manuscript.*

Line 165: I believe you mean "...between case households in the development and validation groups."? Also, can you specify which variables were compared?

*We meant the comparison between the case(+) and control(-) households. The passage has been adequately amended to avoid ambiguity.*

Line 167: It would be helpful to reference the Table 1 as this point so that the reader can figure out the full compliment of 'characteristics' that were compared.

*We have added the suggested table call.*

Line 169: This is the one part of the manuscript that would like to see fleshed out. Specifically, I would like to know how collinearity among ALL variables was assessed and dealt with, particularly given that the discussion identifies that many variables are likely to be colinear. Indeed, results are presented for a collinearity analysis among burrows, runs, and droppings that is not described in the results. It will be important to also establish whether or not there is collinearity among the rat signs and the environmental variables and among the environmental variables. Additionally, it would be helpful to provide more information in the methods on the model selection strategy/metrics and how model fit was assessed. I am particularly interested to know why the more intuitive and parsimonious variable 'any sign of rat infestation' was discarded in favor of a more complex model.

*We used absolute value of correlation coefficients to assess collinearity. We did not identify pairwise correlations exceeding 0.5. We tested a model using the variable 'any sign of rat infestation' but the model had lower performance compared to the one in Table 2. Importantly, we tested the score from this simplified model (as evaluated by a ROC curve) and it had lower sensibility and specificity when compared to the score in Table 3.*

Reviewer #2: The study is well designed especially the case households and control selection.

The rodent marks/runs needs further description especially on how they were identified considering that they could be confused with those of organisms if no further physical evidence was observed. It is worth mentioning where exactly these marks were checked e.g. indoors or outdoors. A more thorough evaluation of rodent presence or activities in an area is that which tiles or the floor is powdered with flour and rodent marks are determined next morning to estimate their presence and activeness in the area. Applying this approach in the two groups of the study cases would give a more reliable evidence.

With regard to predominant rodent species in the area: It is worth mentioning if Rattus rattus which was not recorded in this study through examination of faeces has been previously reported in the area.

Indicate how the faeces of the two species of Rattus (R. norvegicus and R. rattus) can be precisely used to conclude presence of one and absence of the other. This will help clarify the results (Line 220-222) Include in the methodology the abandoned vehicle listed in the supplementary info.

*We have included further rodent description on marks/runs. Those marks were checked only outdoors. We included this information in the text.*

*With regards to a more thorough evaluation, our team has recently developed and validated a track plate method for slum areas that can evaluate rat activity. While this method was not available when the present study was performed (2007-2009) is being used in current longitudinal* epidemiological studies (Hacker 2016; Eyre 2020)

*The shape of fecal pellets is used to identify the species of rodent. Mouse (Size: ¼ inch long; Shape: Small with 1 or both ends pointed), Norway Rat Droppings (Size: ¾ inch long; Shape: Larger, rectangular with blunt ends) and Rattus rattus Droppings (Size: ½ inch long; Shape: Larger, curved, sausage-shaped with pointed ends). We included this information in the text.*

*.. We have not detected* R. rattus*’ droppings in the areas examined (line 232), following the identification guidelines provided by the CDC. Moreover, a later study (Panti-May, 2017) shown that between >700 small mammals trapped during 2013-2014 in slum areas of the same city >95% were Rattus norvegicus*.

*Hacker KP, Minter A, Begon M, Diggle PJ, Serrano S, Reis MG, Childs JE, Ko AI, Costa F. A Comparative Assessment of Track Plates to Quantify Fine Scale Variations in the Relative Abundance of Norway Rats in Urban Slums. Urban Ecosyst. 2016 Jun;19(2):561-575*

*Eyre Max T., Carvalho-Pereira Ticiana S. A., Souza Fábio N., Khalil Hussein, Hacker Kathryn P., Serrano Soledad, Taylor Joshua P., Reis Mitermayer G., Ko Albert I., Begon Mike, Diggle Peter J., Costa Federico and Giorgi Emanuele 2020A multivariate geostatistical framework for combining multiple indices of abundance for disease vectors and reservoirs: a case study of rattiness in a low-income urban Brazilian communityJ. R. Soc. Interface.1720200398*

*Richardson et al. Using fine scale spatial genetics of Norway rats to improve control efforts and reduce leptospirosis risk in urban slum environments October 2016Evolutionary Applications 10(4)*

_______________________________________________________________

Results
-Does the analysis presented match the analysis plan?

-Are the results clearly and completely presented?

-Are the figures (Tables, Images) of sufficient quality for clarity?

Reviewer #1: The results are described very clearly. No suggestions for improvement.

Reviewer #2: Line 220: The methodology do not clearly show how this variable can be used to distinguish between the faecal droppings of the two species, mentioning where these faeces were looked for may help considering that the two species occupy slightly different habitats within human settlements (sewer vs inside houses per se).

*We have included further details on between the faecal droppings of the rodent species (see response to previous comment). All the surveys were performed in the outdoor. Inspections followed the CDC standards stablished by the reference tool applied for the surveys.*

Conclusions
-Are the conclusions supported by the data presented?

-Are the limitations of analysis clearly described?

-Do the authors discuss how these data can be helpful to advance our understanding of the topic under study?

-Is public health relevance addressed?

Reviewer #1: My main comment for the conclusion is that I would be more conservative with regard to describing the predictive power of the tool. Indeed, it is hard to say that the tool 'accurately classified' risk when 42% of case households in the validation group were 'misclassified' (and both sensitivity and specificity are relatively low). Furthermore, it would be beneficial to supplement the discussion in two ways. Firstly, I would like to know why the authors think that such as significant proportion of cases were misclassified according to environmental risk. What risk variables are missing and could the survey be supplemented to capture them? Secondly, it would be beneficial to more explicitly describe appropriate vs inappropriate uses of the tool. This is probably very obvious to the authors but potentially not to future users. For example, the relatively low sensitivity and specificity suggest that the tool may not be appropriate for more precise interventions where accurate household by household identification is needed. However, as the authors note, it can be used as a rough guide to direct existing efforts to maximize efficiency. Can the authors go even one step further and suggest how it could be used in practice? For example, would they recommend that an entire neighborhood be surveyed and interventions be focused on areas where > a specific proportion of households have high scores? Perhaps the utility of neighborhood scores to predict cumulative neighborhood cases could even been an avenue for further study?

*We thank reviewer 1. We have softened the conclusion following reviewer suggestion.*

*Regarding the proportion of households misclassified: the main sources of misclassification in this study were a) the large proportion of households presenting environmental condition which are conductive of leptospirosis transmission (for example in some slums the general household rat infestation rate can be as high as 45.9 [Santos, 2017]); and b) the complex multifactorial transmission dynamic of leptospirosis where not only rats, but environmental factors (water and soil) and social determinants are associated to leptospirosis transmission (see Hagan and Felzemburgh). To improve the score, we are actually performing longitudinal serological studies integrating deep characterization of rat ecology and environmental exposure. We expect that this study design will allow us to better characterize (compared to this case control design) the risk factors for leptospirosis transmission which.*

*Appropriate vs inappropriate uses of the tool: We have extended the paragraph (Lines 329-341) where we explain the use of the score in the context of the city where was developed. In summary, leptospirosis surveillance in Salvador city has identified 11 high risk areas (15% of the area of the city) for the disease. In those areas rodent control is performed 1-3 times per year. Rodent control includes household surveillance for all households in those areas. We propose the use of the score to identify high risk households located inside those high-risk areas (prioritizing rodent chemical control in households with score point values ≥3).*

*Santos et al 2016. Rat infestation associated with environmental deficiencies in an urban slum community with high risk of leptospirosis transmission. Cad. Saúde Pública 2017; 33(2):e00132115*

*Felzemburgh RDM, Ribeiro GS, Costa F, Reis RB, Hagan JE, Melendez AXTO, et al. (2014) Prospective Study of Leptospirosis Transmission in an Urban Slum Community: Role of Poor Environment in Repeated Exposures to the Leptospira Agent. PLoS Negl Trop Dis 8(5): e2927*

*Hagan JE, Moraga P, Costa F, Capian N, Ribeiro GS, Wunder EA Jr, et al. (2016) Spatiotemporal Determinants of Urban Leptospirosis Transmission: Four-Year Prospective Cohort Study of Slum Residents in Brazil. PLoS Negl Trop Dis 10(1): e0004275. https://doi.org/10.1371/journal.pntd.0004275*

Reviewer #2: (No Response)

_______________________________________________________________

Summary and General Comments

Reviewer #1: This is a fantastic paper from a first class team that makes a novel and significant contribution to the field of rat-related research by developing a tool for the prediction of urban leptospirosis based on rat presence and environmental factors. I commend the authors for their elegant execution of the study and for the clarity with which they present their work. I have provided minor comments for improvement above, but overall believe that this paper is highly deserving of publication in this journal.

*We thank reviewer #1 for his/her positive comments.*

In my opinion the one addition that would take this paper to the 'next level' would be the independent modelling of the environmental factors, i.e., without the signs of rat presence.  My concern is that, as the authors point out in the discussion, the colinear nature of the rat presence and many of the included environmental variables means that it is not possible for these variables to be retained in the same model. However, the environmental variables do provide a different piece of the puzzle. For example, they serve as points of intervention for IPM, i.e., the removal of sources of food and harborage vs. reliance on poison baits. Environmental predictors may also require less training and expertise to identify. Finally, given that environmental variables that foster rat infestations are also markers for an 'unhealthy' environment, a focus on these variables would yield resilience-based solutions (i.e., solutions that may protect residents against a plethora of health issues).

*We thank for the recommendation for improving this work to the “next level”. Characteristics of the study design (case-control), sample size restricted to the number of severe cases of leptospirosis and the complex nature of the disease have limited the possibilities for more advanced analysis. We are currently performing this analysis in a larger new set of data (obtained from population based longitudinal cohort studies) which is better design to evaluate larger number of variables (including >80 environmental variables) and hope will promote improvements in the score developed in the current study.*

Ideally, the authors might present both models (with and without rat presence) and compare the fit/predictive power.  Overall, I don't think that this analysis (or the lack of it) should be a barrier to proceeding with publication. It is primarily a suggestion of what I would like to see in the literature and what might make this study even more impactful.

*We thank the reviewer, and we prefer to not include the new set of analysis as the reviewer indicated they are not essential. As explained in the previous comment, we believe that study design and sample size preclude the proper answer to this comment which will be tackled in an upcoming work specially designed for this research question.*
