# Supplementary material for: Household rat infestation in urban slum populations: Development and validation of a predictive score for leptospirosis
Source: PLoS Negl Trop Dis. 2021 Mar 3;15(3):e0009154. doi: 10.1371/journal.pntd.0009154 (PMC7959339; doi:10.1371/journal.pntd.0009154)
Supplement: Attachment — Submitted filename: Dear editors.docx [file pntd.0009154.s003.docx]

Dear editors, PLoS Neglected Tropical Diseases.

Here, we submit our revised manuscript "Household Rat Infestation in Urban Slum Populations: Development and Validation of a Predictive Score for Leptospirosis". We incorporated the latest suggested changes, as well as request further instructions on a final comment, as detailed below.

Sincerely,

The authors.

1. Line 115: suggest replacing "with" with "including" as it is clearer that the most prevalent species was one of a group tested, as opposed to the only serovar/species tested.

*Response: changes made as suggested.*

2. Line 198: suggest replacing "laboratorial confirmed" with "laboratory-confirmed".

*Response: changes made as suggested.*

3. We did not see a reply to our request to make sure data (and ideally code) are available to reproduce analyses, as required by the PLoS Data Policy. Please include these data, with an explanation of what exactly is included, in the revised submission so we are able to proceed.

*Response: As signaled on the first submission of this manuscript, the data is not available at this moment for public sharing, but it was arranged that it would be deposited at the end of the publication process as following the submission form instructions. So far, the instructions we received on the matter are as disposed below, on an e-mail sent on the occasion of the submission of the first revised version:*

*“*You have indicated an exemption from our data availability policy in your Data Availability Statement. This has been passed to the Editor for their consideration and approval. One of the team will be in touch with you separately if there are any issues. Please be assured that this assessment of your exemption will not hold up the peer review process.”

*Given that, we would like to know how to proceed in this specific step of the submission, as we are yet to receive the communication from the team regarding the submission of the data for a repository.*
